# Heritability of brain volume on MRI in middle to advanced age: A twin study of Japanese adults

**DOI:** 10.1371/journal.pone.0175800

**Published:** 2017-04-20

**Authors:** Matthew W. Lukies, Yoshiyuki Watanabe, Hisashi Tanaka, Hiroto Takahashi, Soshiro Ogata, Kayoko Omura, Shiro Yorifuji, Noriyuki Tomiyama, the Osaka University Twin Research Group

**Affiliations:** 1 Department of Diagnostic and Interventional Radiology, Osaka University Graduate School of Medicine, Suita, Japan; 2 Department of Health Promotion Science, Osaka University Graduate School of Medicine, Suita, Japan; 3 Osaka University Twin Research Group, Osaka University Graduate School of Medicine, Suita, Japan; 4 Department of Public Health and Community Nursing, Mie Prefectural Nursing College, Mie, Japan; 5 Division of Functional Diagnostic Science, Osaka University Medical School, Suita, Japan; University of Modena and Reggio Emilia, ITALY

## Abstract

Brain atrophy is part of the aging process and accelerated by neurodegenerative diseases, so an understanding of the background heritability of brain volume is essential. The purpose of this study was to determine the heritability of brain volume in middle to advanced age East Asian adults, an age group less studied and an ethnicity not previously studied. 3T magnetic resonance images were obtained and volumetric analyses conducted for a total of 74 individuals, 20 monozygotic twin pairs (mean age 61y min 41y max 75y) and 17 dizygotic twin pairs (mean age 64y min 41y max 85y). Total brain volume and a further seven regions were assessed, including lobar volumes, lateral divisions, and separated grey and white matter. Additive genetics and unique environment (AE) models for global brain volumes including total brain (90%), grey matter (91%) and white matter (84%) and many lobar volumes demonstrated high heritability in our study population. Our results present the heritability of brain volume in middle to advanced age as possibly higher in East Asian adults.

**Highlights**

- Twin study of brain volume measurements on 3.0 T high resolution MRI
- East Asian middled-aged and older adults
- Total brain, grey matter, white matter and lobar volumes decrease with age
- Total brain (90%), grey matter (91%), white matter (84%) and lobar volumes have strong heritability

## Introduction

Brain volume is dynamic, and known to change over time including during childhood, adulthood and later life [1–5], and such changes have been found to have a contribution from genetics [1, 6]. Whilst brain volume appears to be relatively stable between 20 and 40 years of age, as we enter late middle and advanced age, brain volume gradually decreases over time [7, 8]. Brain atrophy is a common finding on cranial imaging and is part of the normal aging process in healthy individuals [4, 7, 8], but accelerated atrophy is associated with cognitive decline and diseases such as dementia [1, 9–12]. Understanding the background heritability of brain volume in middle-aged and older adults is essential for investigating diseases associated with brain atrophy in this age group and determining its usefulness as a diagnostic finding on MRI.

Human brain volume is known to be highly heritable, with heritability estimates for total brain volume between 46% and over 90% for paediatric, middle and advanced age populations [13–23]. Heritability of brain volume in middle-aged and older adults is less studied, but previous estimates for total brain volume range between 63% [21] and 81% [6, 24]. Furthermore, whilst there have been studies of brain volume volumetrics and temporal change [4, 5] in an East Asian population, there have been no studies, to our knowledge, that calculated brain heritability in middle-aged to older adults of this ethnicity.

Our study firstly examines MRI-derived volumetric measurements of the global and lobar brain, including the influence of age, gender and laterality, followed by calculation of heritability of global and lobar brain volume measurements using a classical twin design for middle to advanced age Japanese male and female twins.

## Methods

### Subjects

The Osaka University Twin Registry [25] comprises a total of 259 monozygotic and 39 dizygotic twin pairs. Written informed consent was obtained from all subjects after explaining the purpose and possible consequences of the study, and the study was approved by the ethics committee of Osaka University Graduate School of Medicine (No. 10190). Zygosity was confirmed using 15 loci short tandem repeat (STR) markers with complete concordance of these STRs diagnostic of a monozygotic twin pair. Of these twins, 45 monozygotic and 18 dizygotic pairs were recruited for MRI brain as part of neuroscientific research. As a subset for this study of the heritability of brain volume in middle to advanced age, 74 twins over 40 years old were selected from the database and were age and sex matched, comprising 20 monozygotic twin pairs and 17 dizygotic twin pairs. The monozygotic twin pairs consisted of 10 male-male pairs and 10 female-female pairs, and the dizygotic twin pairs consisted of 8 male-male pairs, 8 female-female pairs and 1 male-female pair (see Table 1).

**Table 1:** Age, gender and MMSE (mini-mental state examination) of twin participants.

	All	Monozygotic	Dizygotic
Gender	37 M, 37 F	20 M, 20 F	17 M, 17 F
Age mean (SD) [range]	62.5 y (10.2) [41 to 85]	61.0 y (8.70) [42 to 75]	64.1 y (13.3) [41 to 85]
MMSE mean (SD) [range]	28.1 (1.74) [22 to 30]	28.3 (1.80) [22 to 30]	27.8 (1.67) [22 to 30]

On average, the 74 participants were 62.5 years old, ranging between 41 years and 85 years, at the time of the MRI scan. The monozygotic twin pairs had a mean age of 61.0 years, ranging from 41 years to 75 years old. The dizygotic twin pairs had a mean age of 64.1 years, ranging from 41 years to 85 years old. Of the 74 participants, 68 completed a mini-mental state examination (MMSE) questionnaire. The overall average score for all participants together was 28.1, ranging from 22 to 30, and there was no significant difference based on zygosity.

### Brain imaging & processing

MRI images were obtained using two different scanners, due to equipment changes, at a single centre, the Department of Diagnostic and Interventional Radiology, Osaka University Hospital, Japan. 10 participants were scanned on a General Electric (GE) Signa HDxt 3.0 Tesla MRI scanner and sagittal images obtained; time to echo (TE) 2.9 msec, time to repetition (TR) 7.0 msec, inversion time = 400 msec, in-plane resolution = 1x1 mm, slice thickness 1 mm, slice number 180, flip angle 11. The remaining 64 participants were scanned on a Philips Achieva 3.0 Tesla TX MRI scanner and sagittal images obtained; TE 3.1 msec, TR 6.7 msec, inversion time 880 msec, in-plane resolution = 1x1 mm, slice thickness 1 mm, slice number 180, flip angle 8. Both individuals of a twin pair were scanned with the same MRI scanner on the same day.

3D T1 volume images were exported as dicom files and then converted to FSL 4D nifty files using the software MRIcron. Image segmentation was performed using SPM12 software (University College London) Diffeomorphic Anatomical Registration Through Exponentiated Lie Algebra (DARTEL) toolbox, WFU Pickatlas version 2.5 [26] and Talairach Daemon Human Atlas [27] in Matlab. Masks produced by the WFU Pickatlas were adjusted to match the voxel size of the DARTEL generated images by using the Coregister (Reslice) function in SPM12. Maps for each lobar region were visually evaluated to confirm close alignment with the DARTEL generate images. Total brain volume and a total of seven regions of interest were mapped and corrected for total intracranial volume (ICV) (V_2_ = V_1_ × mean ICV / ICV; where V_2_ = corrected volumetric measure, V_1_ = original volumetric value), including global volumes, the four cerebral lobes, cerebellum and brain stem, as well as subdivided measurements for left and right hemispheres, and grey and white matter.

### Statistical analyses

The mean and standard deviation for each brain volume measurement were calculated from the amalgamated volume data. Using mixed effects linear regression models in statistical platform R (version 3.2.2), the correlation with age, and differences based on gender, laterality, scanner and zygosity were then assessed and compared. Significant difference in volume measurements based on gender was identified in our study group, so the one male-female dizygotic twin pair was excluded from further twin analyses.

### Twin analyses

As an initial step, the intra-twin correlations were calculated and compared for the monozygotic and dizygotic twin groups using statistical platform R. A higher correlation for monozygotic twins compared with dizygotic twins provides a preliminary indication that there is likely to be a significant influence of genetic factors. In addition to this, the volume differences within monozygotic and dizygotic twin pairs were calculated and compared, including correlation with age. A relative similarity of monozygotic twin measurements compared to dizygotic is also a preliminary indicator of the influence of genetic factors.

Classical twin studies use the assumption that monozygotic twins share 100% of their segregating genes, where as dizygotic twins share 50% [28]. The twin analyses performed in this study with structural equation modelling followed this underlying assumption.

Structural equation models were constructed using OpenMx (version 2.3.1) in statistical platform R (version 3.2.2), using a customised code based on that originally written by Hermine Maes (Virginia Institute for Psychiatric and Behavior Genetics, Virginia Commonwealth University). Four models, ACE, AE, CE and E, were produced, involving the following phenotype variance components: additive genetic effects (A), common environmental effects (C) and unique environmental effects (E). The alternative models were then compared for each volume measurement based on fit and simplicity using the Akaike information criterion (AIC), likelihood ratios and p-values. The procedure for choosing the best model fit was the lowest AIC, p-value > 0.05, and likelihood ratio χ^2^ test.

**Fig 1:**
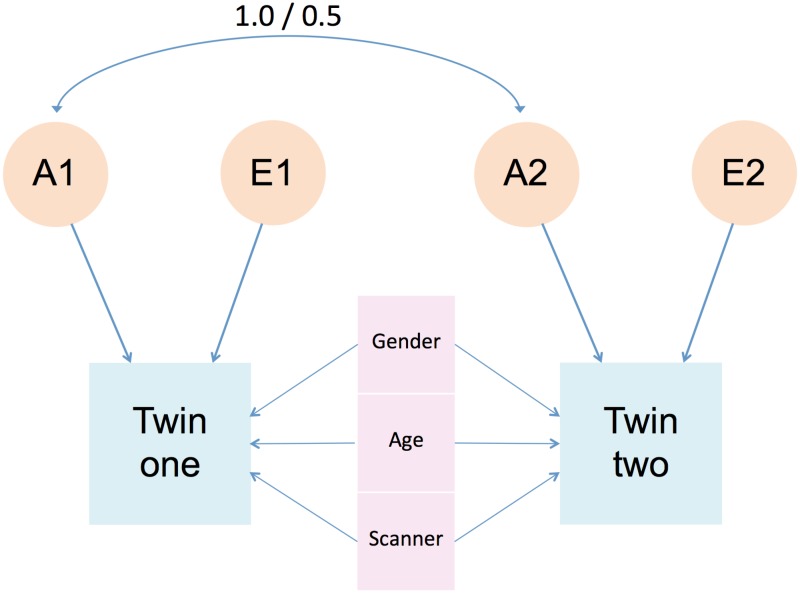
Path diagram representing the structural equation model used to calculate A (additive genetics) and E (unique environmental) factors.

Separate hemispheric volumes were mapped and analysed separately to compare the difference in genetic and environmental factors based on laterality. To control for the variation in age, gender and scanner, these factors were included in the structural equation models as covariates (Fig 1). To estimate the influence of the covariates, age and gender, on A, we repeated the AE model correcting only for the other covariates (e.g. scanner and gender if estimating the influence of age) then calculated the path between that tested covariate and A. Path estimates were considered to be possibly significant if the 95% confidence interval did not cross zero.

There were two individuals in the analysis with an MMSE score of below 24 (one dizygotic twin with a score of 22 and one monozygotic twin with a score of 22). Intra-pair correlations and heritability models for total brain, grey matter and white matter volumes with these individuals and their corresponding twin partners excluded were repeated and compared to the results with these two twin pairs included. Intra-pair correlations and heritability estimates (A) closely aligned with the values for the overall analysed group, with widely overlapping confidence intervals.

## Results

### Intracranial volumetric analyses

**Fig 2:**
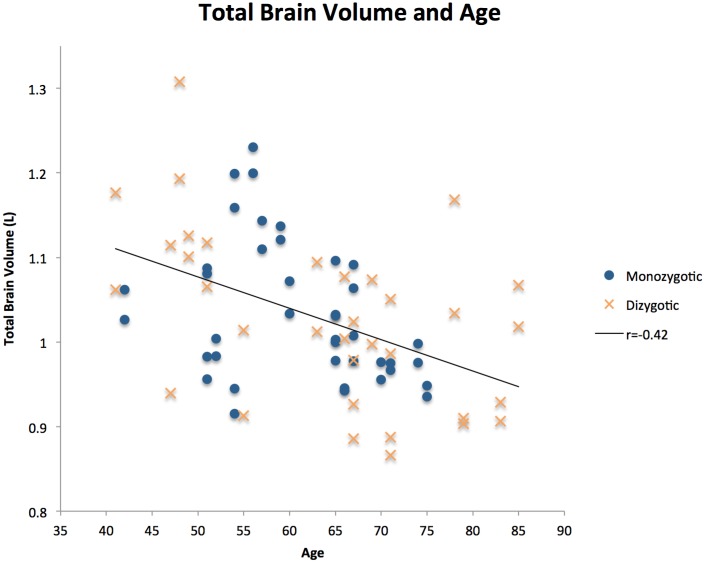
Plot of total brain volume and age.

Table 2 displays the results from volumetric analyses of the measurements of global and lobar brain volumes. Where p≥0.05, the symbol—is written; where p<0.05 the symbol * is written; where p<0.01 the symbol ** is written. The mean total brain volume for all twins was 1034 cm^3^ with a standard deviation of 90.2. Fig 2 illustrates the correlations between total brain volume and age.

**Table 2:** Volumetric analyses.

Region	Volume	Mean	SD	Laterality	Gender Difference (M—F)		Age Correlation
Brain	Total	1034	90.2		M>F*	48	-0.42**
	Right	514.2	47.3	R>L*	-		-0.44**
	Left	508.4	41.9		M>F*	21	-0.44**
	TGM	599.8	71.1		-		-0.50**
	RGM	296.2	33.7	R>L**	-		-0.51**
	LGM	292.3	33.8		-		-0.51**
	TWM	434.5	49.8		M>F**	28	-0.047**
	RWM	218.0	29.3	-	-		-0.043
	LWM	216.0	24.5		M>F**	14	-0.041
	CSF	354.0	99.0		M>F**	12	0.64**
Cerebrum	Total	892.7	74.4		M>F*	37	-0.44**
	Right	446.8	37.0	R>L**	M>F*	17	-0.44**
	Left	441.7	37.1		M>F*	20	-0.43**
	TGM	504.5	60.6		-		-0.51**
	RGM	253.5	30.2	R>L**	-		-0.51**
	LGM	248.2	29.9		-		-0.51**
	TWM	388.2	45.3		M>F**	26	-0.042
	RWM	193.3	22.6	-	M>F**	12	-0.049
	LWM	193.5	22.7		M>F**	14	-0.030
Cerebellum	Total	109.8	9.76		-		-0.29*
	Right	54.33	4.98	L>R**	-		-0.27*
	Left	55.42	5.09		-		-0.30*
	TGM	83.25	8.79		-		-0.41**
	RGM	41.03	4.47	L>R**	-		-0.38**
	LGM	42.21	4.62		-		-0.40**
	TWM	26.51	4.24		M>F*	1.9	-0.017
	RWM	13.30	2.14	-	-		-0.016
	LWM	13.21	2.14		M>F**	1.0	-0.016
Frontal	Total	247.2	36.3		M>F**	28	-0.31**
	Right	124.8	19.0	-	M>F**	14	-0.25*
	Left	122.3	19.3		M>F**	14	-0.34**
	TGM	135.5	23.9		-		-0.44**
	RGM	68.46	12.3	R>L*	-		-0.40**
	LGM	67.08	12.2		-		-0.46**
	TWM	111.6	19.0		M>F**	19	-0.037
	RWM	56.38	10.3	-	M>F**	9.3	-0.020
	LWM	55.27	10.0		M>F**	9.8	-0.096
Temporal	Total	171.7	19.5		M>F**	15	-0.35**
	Right	87.62	9.56	R>L**	M>F**	6.9	-0.36**
	Left	84.07	10.1		M>F**	8.4	-0.34**
	TGM	107.0	12.9		M>F**	6.6	-0.48**
	RGM	54.19	6.29	R>L**	M>F*	2.7	-0.48**
	LGM	52.80	6.70		M>F**	3.8	-0.47**
	TWM	64.70	10.7		M>F**	8.8	-0.061
	RWM	33.43	5.47	R>L**	M>F**	4.2	-0.066
	LWM	31.27	5.38		M>F**	4.6	-0.054
Parietal	Total	120.5	16.2		M>F**	13	-0.0023
	Right	60.22	8.02	-	M>F**	6.4	-0.013
	Left	60.30	8.50		M>F**	6.8	-0.0075
	TGM	69.35	10.2		-		-0.30*
	RGM	35.00	5.15	R>L**	-		-0.30**
	LGM	34.34	5.19		-		-0.29*
	TWM	51.18	11.9		M>F**	9.5	-0.026*
	RWM	25.22	5.82	L>R**	M>F**	4.6	-0.025*
	LWM	25.96	6.18		M>F**	4.9	-0.026*
Occipital	Total	90.57	12.4		-		-0.052
	Right	43.71	6.73	L>R**	-		-0.070
	Left	46.80	7.87		-		-0.028*
	TGM	55.91	7.91		-		-0.11
	RGM	27.90	4.47	-	-		-0.19
	LGM	27.96	4.71		-		-0.010
	TWM	34.66	8.39		-		-0.043**
	RWM	15.80	3.91	L>R**	-		-0.034**
	LWM	18.84	5.10		-		-0.044**
Brainstem	Total	22.14	3.15		-		-0.57**
	Right	10.93	1.60	L>R**	-		-0.56**
	Left	11.21	1.58		-		-0.57**

T = total, R = right, L = left, GM = grey matter, WM = white matter, CSF = cerebrospinal fluid. Volumes are adjusted for ICV and expressed in cm^3^;

- indicates p≥0.05;

* indicates p<0.05;

** indicates p<0.01.

### Laterality and gender differences

Right brain hemisphere volume was, on average, slightly larger than left. Volume measurements for the cerebrum and temporal lobe were also significantly larger on the right compared to the left, where as those for the cerebellum, occipital lobe and brainstem were greater on the left side. Grey matter volumes were generally larger on the right side compared to left, with the exception of the cerebellum and occipital lobe. Volume measurements of white matter were not significantly different depending on laterality for the global measurements of total volume, cerebrum and cerebellum, but were different for several lobes, with either the left or right side being larger.

The majority of cerebral volume measurements (adjusted for ICV) were significantly larger in males compared to females, with the exception of the occipital lobe. Also, the cerebellar and brainstem volumes did not demonstrate significant difference based on gender.

### Age correlations

The correlations with age for total brain volume and the seven lobar regions are included in Table 2. Most regions including total brain and lobar volumes demonstrated a significant negative correlation with age, with the exception of CSF. CSF was significantly positively correlated with age. Total grey matter demonstrated a stronger negative correlation compared to white matter. Lobar grey matter volumes, with the exception of occipital, also demonstrated stronger negative age correlation when compared to white matter volumes.

### Within-pair analyses

**Fig 3:**
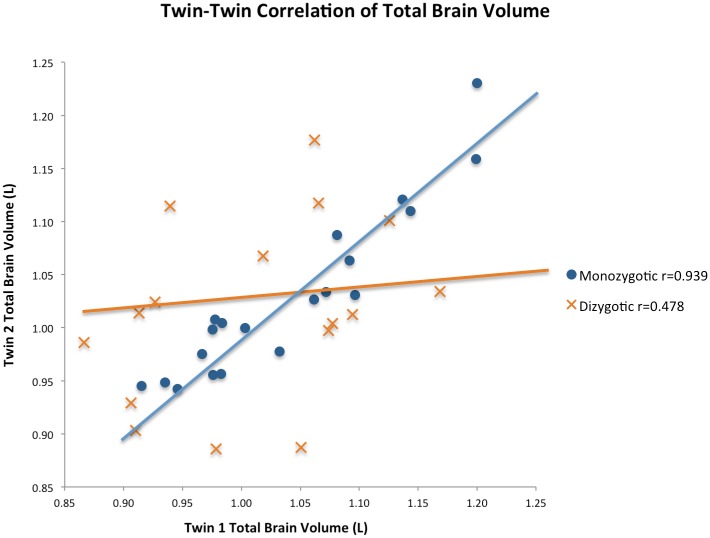
Twin-twin correlation plot of total brain volume.

The intra-pair differences and correlations are presented in Table 3. The volume measurements for monozygotic twins were significantly more congruent based on intra-pair difference for all areas studied, with the exception of left sided occipital lobe measurements, compared to dizygotic twins. There were strong and significant intra-pair correlations for all volume measurements in monozygotic twins (Fig 3). The calculated correlation coefficient was highest (over 0.90) for larger volume measurements such as total brain, total grey matter, cerebrum, frontal and temporal lobes. 26 of the volume measurements demonstrated significant correlation in dizygotic twins, whilst 41 did not. The correlation coefficients for dizygotic twins were lower than for monozygotic twins and included wider confidence intervals, frequently approaching or crossing zero. The within-pair differences in total brain, cerebrum, cerebellum, frontal lobe, temporal lobe, parietal lobe, occipital lobe and brainstem volumes all demonstrated positive correlation with age (r = 0.30, 0.28, 0.25, 0.14, 0.26, 0.38, 0.20 and 0.17 respectively). These correlations, however, were non-significant (p≥0.05) except for temporal lobe volume, where p = 0.02. This suggests that increasing age may introduce more environmental factors.

**Table 3:** Twin intra-pair analyses.

		Mean Intra-pair Difference			Intra-pair Correlation			
Region	Volume	Monozygotic	Dizygotic	Sig. Diff.	Monozygotic	95% CI	Dizygotic	95% CI
Brain	Total	25.7	93.4	**	0.939**	0.85, 0.98	0.478	-0.023, 0.79
	Right	13.1	53.7	**	0.923**	0.81, 0.97	0.424	-0.091, 0.76
	Left	12.5	43.4	**	0.919**	0.80, 0.97	0.487	-0.012, 0.79
	TGM	19.8	54.5	**	0.955**	0.89, 0.98	0.647**	0.22, 0.87
	RGM	10.1	26.0	**	0.936**	0.84, 0.97	0.653**	0.23, 0.87
	LGM	10.4	25.0	**	0.935**	0.84, 0.97	0.667**	0.26, 0.87
	TWM	19.7	45.1	**	0.871**	0.70, 0.95	0.358	-0.17, 0.73
	RWM	9.39	31.6	**	0.884**	0.73, 0.95	0.127	-0.39, 0.59
	LWM	10.4	22.0	**	0.851**	0.65, 0.94	0.366	-0.16, 0.73
	CSF	30.4	49.7	**	0.866**	0.69, 0.95	0.876**	0.67, 0.96
Cerebrum	Total	21.9	79.5	**	0.927**	0.82, 0.97	0.482	-0.018, 0.79
	Right	11.6	39.9	**	0.923**	0.81, 0.97	0.496	0.00030, 0.80
	Left	11.1	39.0	**	0.924**	0.81, 0.97	0.462	-0.043, .78
	TGM	17.8	45.5	**	0.942**	0.86, 0.98	0.666**	0.25, 0.87
	RGM	9.46	23.0	**	0.933**	0.83, 0.97	0.665**	0.25, 0.87
	LGM	8.81	22.0	**	0.946**	0.87, 0.98	0.659**	0.24, 0.87
	TWM	17.7	41.7	**	0.878**	0.71, 0.95	0.339	-0.19, 0.71
	RWM	8.33	20.8	**	0.896**	0.75, 0.96	0.332	-0.20, 0.71
	LWM	9.54	20.8	**	0.855**	0.66, 0.94	0.348	-0.18, 0.72
Cerebellum	Total	3.17	9.26	**	0.881**	0.72, 0.95	0.394	-0.13, 0.74
	Right	2.28	5.13	**	0.823**	0.60, 0.93	0.342	-0.18, 0.72
	Left	2.38	4.45	**	0.788**	0.53, 0.91	0.445	-0.066, 0.77
	TGM	3.23	7.57	**	0.884**	0.72, 0.95	0.448	-0.062, 0.77
	RGM	1.93	4.07	**	0.844**	0.64, 0.94	0.425	-0.089, 0.76
	LGM	2.20	3.74	*	0.754**	0.47, 0.90	0.466	-0.039, 0.78
	TWM	1.72	2.78	**	0.870**	0.70, 0.95	0.733**	0.37, 0.90
	RWM	0.933	1.46	**	0.843**	0.64, 0.94	0.697**	0.31, 0.89
	LWM	0.863	1.37	*	0.855**	0.66, 0.94	0.753**	0.41, 0.91
Frontal	Total	12.6	33.5	**	0.922**	0.81, 0.97	0.483	-0.017, 0.79
	Right	9.07	13.6	*	0.826**	0.61, 0.93	0.573*	0.11, 0.83
	Left	7.38	20.3	**	0.926**	0.82,0.97	0.302	-0.23, 0.69
	TGM	7.57	18.1	**	0.937**	0.85, 0.98	0.667**	0.26, 0.87
	RGM	5.07	8.14	**	0.864**	0.68, 0.95	0.709**	0.33, 0.89
	LGM	4.01	10.8	**	0.956**	0.89, 0.98	0.566*	0.10, 0.83
	TWM	6.28	18.8	**	0.912**	0.79, 0.97	0.346	-0.18, 0.72
	RWM	4.25	7.90	**	0.834**	0.62, 0.93	0.485	-0.014, 0.79
	LWM	4.25	11.1	**	0.880**	0.72, 0.95	0.119	-0.40, 0.58
Temporal	Total	4.63	19.0	**	0.954**	0.89, 0.98	0.433	-0.08, 0.76
	Right	2.92	9.28	**	0.923**	0.81, 0.97	0.409	-0.11, 0.75
	Left	2.70	9.69	**	0.954**	0.88, 0.98	0.454	-0.054, 0.78
	TGM	4.27	10.6	**	0.943**	0.86, 0.98	0.494	-0.0017, 0.80
	RGM	2.48	5.00	**	0.910**	0.78, 0.96	0.449	-0.060, 0.77
	LGM	2.14	5.70	**	0.949**	0.87, 0.98	0.517*	0.028, 0.81
	TWM	3.49	9.02	**	0.922**	0.81, 0.97	0.431	-0.082, 0.76
	RWM	1.61	4.76	**	0.922**	0.81, 0.70	0.404	-0.11, 0.75
	LWM	2.24	4.27	**	0.882**	0.72, 0.95	0.459	-0.047, 0.78
Parietal	Total	5.55	16.6	**	0.896**	0.75, 0.96	0.355	-0.17, 0.72
	Right	3.02	8.80	**	0.857**	0.67, 0.94	0.359	-0.17, 0.73
	Left	3.31	8.08	**	0.869**	0.69, 0.95	0.331	-0.20, 0.71
	TGM	4.44	8.91	**	0.888**	0.73, 0.96	0.594*	0.14, 0.84
	RGM	2.15	4.76	**	0.861**	0.68, 0.94	0.589*	0.13, 0.84
	LGM	2.38	4.40	**	0.866**	0.69, 0.95	0.574*	0.11, 0.83
	TWM	3.67	9.60	**	0.923**	0.81, 0.97	0.383	-0.14, 0.74
	RWM	2.12	4.85	**	0.888**	0.73, 0.96	0.362	-0.16, 0.73
	LWM	2.31	4.95	**	0.904**	0.77, 0.96	0.394	-0.13, 0.74
Occipital	Total	5.94	12.7	**	0.757**	0.47, 0.90	0.495	-0.00066, 0.80
	Right	4.16	8.91	**	0.561*	0.16, 0.80	0.017	-0.51, 0.48
	Left	5.04	5.17	-	0.626**	0.25, 0.84	0.708**	0.33, 0.89
	TGM	3.34	6.72	**	0.743**	0.45, 0.85	0.557*	0.085, 0.83
	RGM	2.07	5.33	**	0.650**	0.29, 0.85	0.103	-0.41, 0.57
	LGM	3.05	3.14	-	0.563**	0.16, 0.81	0.635**	0.20, 0.86
	TWM	3.42	6.20	**	0.834**	0.62, 0.93	0.659**	0.24, 0.87
	RWM	2.43	3.87	**	0.666**	0.32, 0.86	0.248	-0.28, 0.66
	LWM	2.45	2.56	-	0.760**	0.48, 0.90	0.804**	0.51, 0.93
Brainstem	Total	1.34	2.40	**	0.838**	0.63, 0.93	0.693**	0.30, 0.88
	Right	0.700	1.22	**	0.843**	0.64, 0.94	0.663**	0.25, 0.87
	Left	0.693	1.20	**	0.806**	0.57, 0.92	0.706**	0.32, 0.89

T = total, R = right, L = left, GM = grey matter, WM = white matter, CSF = cerebrospinal fluid. Volumes are adjusted for ICV and expressed in cm^3^;

- indicates p≥0.05;

* indicates p<0.05;

** indicates p<0.01.

Intra-pair correlations for total brain, grey and white matter volume with exclusion of the two individuals with MMSE of 22 and their corresponding twin partners closely aligned with the values for the overall analysed group (0.938**, 0.955** and 0.871** respectively for monozygotic pairs; 0.426, 0.601* and 0.353 respectively for dizygotic pairs).

### Twin analyses and heritability estimates

Results from the structural equation models including A, C and E factors where relevant are presented in Table 4. Based on AIC, log-likelihood ratios, and the likelihood ratio χ^2^ test, the majority of volume measurements studied fit best with an AE model, supporting a significant influence from genetic factors. CSF, left cerebellar white matter and left sided occipital measurements fit best with a CE model, indicating a strong influence of common and unique environmental factors in these areas.

**Table 4:** Structural equation modelling results.

									Influence of covariate on A	
Region	Volume	Model	A	95% CI	C	95% CI	E	95% CI	Gender	Age
Brain	Total	AE	0.902	0.78, 0.95			0.0980	0.047, 0.22	-0.022	-0.028
	Right	AE	0.881	0.73, 0.94			0.119	0.057, 0.28	-0.023	-0.034
	Left	AE	0.861	0.68, 0.93			0.139	0.067, 0.32	-0.035*	-0.045*
	TGM	AE	0.912	0.81, 0.96			0.0878	0.043, 0.19	-0.013	-0.030
	RGM	AE	0.874	0.87, 0.94			0.126	0.062, 0.13	-0.015	-0.045
	LGM	AE	0.865	0.71, 0.93			0.135	0.066, 0.29	-0.019	-0.051
	TWM	AE	0.843	0.66, 0.92			0.158	0.077, 0.34	-0.027	-0.011
	RWM	AE	0.872	0.70, 0.94			0.128	0.061, 0.30	-0.013	-0.0070
	LWM	AE	0.817	0.61, 0.91			0.183	0.089, 0.39	-0.032	-0.011
	CSF	CE			0.681	0.46, 0.82	0.319	0.18, 0.54		
Cerebrum	Total	AE	0.875	0.71, 0.94			0.126	0.060, 0.29	-0.032	-0.042
	Right	AE	0.870	0.70, 0.94			0.130	0.062, 0.30	-0.033	-0.044*
	Left	AE	0.869	0.69, 0.94			0.131	0.062, 0.31	-0.035*	-0.045*
	TGM	AE	0.883	0.75, 0.94			0.117	0.058, 0.25	-0.015	-0.044
	RGM	AE	0.868	0.72, 0.94			0.132	0.065, 0.28	-0.016	-0.048
	LGM	AE	0.889	0.76, 0.95			0.111	0.055, 0.24	-0.016	-0.041
	TWM	AE	0.850	0.67, 0.93			0.150	0.073, 0.33	-0.026	-0.0098
	RWM	AE	0.873	0.72, 0.94			0.127	0.061, 0.28	-0.022	-0.0090
	LWM	AE	0.822	0.61, 0.91			0.178	0.087, 0.39	-0.032	-0.010
Cerebellum	Total	AE	0.846	0.67, 0.93			0.154	0.075, 0.34	-0.019	-0.024
	Right	AE	0.773	0.52, 0.89			0.227	0.11, 0.48	-0.027	-0.035
	Left	AE	0.721	0.45, 0.86			0.279	0.14, 0.55	-0.038	-0.045
	TGM	AE	0.812	0.59, 0.91			0.188	0.090, 0.41	-0.018	-0.043
	RGM	AE	0.753	0.48, 0.88			0.247	0.12, 0.52	-0.020	-0.053*
	LGM	AE	0.605	0.27, 0.80			0.395	0.20, 0.73	-0.041	-0.094*
	TWM	AE	0.823	0.66, 0.91			0.177	0.092, 0.34	-0.021	-0.00070
	RWM	AE	0.786	0.59, 0.89			0.214	0.11, 0.41	-0.024	-0.00090
	LWM	CE			0.749	0.57, 0.86	0.251	0.14, 0.43		
Frontal	Total	AE	0.867	0.69, 0.94			0.133	0.063, 0.31	-0.031	-0.028
	Right	AE	0.749	0.50, 0.87			0.251	0.13, 0.50	-0.045*	-0.040
	Left	AE	0.862	0.63, 0.94			0.138	0.062, 0.37	-0.037	-0.036
	TGM	AE	0.870	0.79, 0.94			0.130	0.064, 0.21	0.015	-0.037
	RGM	AE	0.759	0.53, 0.88			0.241	0.12, 0.47	-0.022	-0.057*
	LGM	AE	0.907	0.78, 0.96			0.0927	0.044, 0.22	-0.012	-0.028
	TWM	AE	0.877	0.71, 0.94			0.123	0.059, 0.29	-0.034	-0.0095
	RWM	AE	0.783	0.55, 0.89			0.217	0.11, 0.45	-0.050	-0.010
	LWM	AE	0.808	0.48, 0.92			0.192	0.084, 0.52	-0.065*	-0.025
Temporal	Total	AE	0.923	0.82, 0.96			0.0772	0.0369, 0.183	-0.021	-0.019
	Right	AE	0.870	0.69, 0.94			0.130	0.061, 0.31	-0.036	-0.034
	Left	AE	0.920	0.81, 0.96			0.0801	0.038, 0.19	-0.023	-0.020
	TGM	AE	0.886	0.73, 0.95			0.114	0.054, 0.27	-0.025	-0.040
	RGM	AE	0.819	0.57, 0.92			0.181	0.084, 0.43	-0.037	-0.066*
	LGM	AE	0.895	0.75, 0.95			0.105	0.050, 0.25	-0.025	-0.037
	TWM	AE	0.899	0.78, 0.95			0.101	0.049, 0.22	-0.021	-0.0059
	RWM	AE	0.902	0.78, 0.95			0.0984	0.048, 0.22	-0.019	-0.0059
	LWM	AE	0.844	0.66, 0.92			0.156	0.077, 0.34	-0.034	-0.0086
Parietal	Total	AE	0.865	0.70, 0.94			0.135	0.065, 0.31	-0.031	-0.010
	Right	AE	0.806	0.57, 0.91			0.194	0.093, 0.43	-0.050*	-0.018
	Left	AE	0.831	0.62, 0.92			0.169	0.081, 0.38	-0.038	-0.011
	TGM	AE	0.846	0.68, 0.92			0.154	0.078, 0.32	-0.018	-0.027
	RGM	AE	0.809	0.61, 0.90			0.191	0.096, 0.39	-0.022	-0.035
	LGM	AE	0.820	0.63, 0.91			0.180	0.0909, 0.37	-0.020	-0.029
	TWM	AE	0.888	0.74, 0.95			0.112	0.054, 0.26	-0.026	-0.00030
	RWM	AE	0.827	0.60, 0.92			0.173	0.082, 0.40	-0.044	-0.00070
	LWM	AE	0.861	0.68, 0.93			0.139	0.067, 0.32	-0.032	0.00010
Occipital	Total	AE	0.643	0.38, 0.82			0.357	0.18, 0.62	-0.030	0.000
	Right	AE	0.241	0.00, 0.61			0.759	0.39, 1.0	-0.073	-0.031
	Left	CE			0.559	0.30, 0.75	0.441	0.26, 0.70		
	TGM	AE	0.689	0.41, 0.84			0.311	0.16, 0.58	-0.024	-0.036
	RGM	AE	0.449	0.090, 0.74			0.551	0.26, 0.99	-0.063	-0.13
	LGM	CE			0.564	0.42, 0.75	0.437	0.25, 0.58		
	TWM	AE	0.724	0.49, 0.86			0.276	0.14, 0.51	-0.011	-0.026
	RWM	AE	0.455	0.050, 0.73			0.545	0.27, 0.95	-0.023	-0.031
	LWM	CE			0.642	0.41, 0.80	0.359	0.20, 0.59		
Brainstem	Total	AE	0.742	0.51, 0.87			0.258	0.14, 0.49	-0.0070	-0.048
	Right	AE	0.755	0.52, 0.87			0.245	0.13, 0.47	-0.0050	-0.045
	Left	AE	0.693	0.44, 0.834			0.307	0.16, 0.56	-0.0099	-0.055

The best fit model, either AE (Additive genetics & unique environment) and CE (common environment & unique environment) are shown according to best fit based on AIC, log-likelihood ratios and p-values; influence of the covariate is expressed as the path between the covariate and A using AE models corrected only for ICV and scanner. Path estimates with possible significance are marked with * (confidence intervals not crossing zero). T = total, R = right, L = left, GM = grey matter, WM = white matter, CSF = cerebrospinal fluid.

The gross measurements of total brain, grey and white matter volume demonstrated high levels of heritability of 0.902, 0.912 and 0.843 respectively. The highest heritability estimate amongst the lobar volumes was 0.923 for total temporal volume. Total cerebellar, frontal and parietal volumes also demonstrated high heritability with estimates of 0.846, 0.867 and 0.865 respectively. A more moderate contribution of genetic factors was observed for the occipital lobe and brainstem. Total occipital lobe volume heritability was calculated as 0.643, however the three left sided measurements (total, grey and white matter) fit best with a CE model. Total brainstem heritability was estimated as 0.742, a moderate result compared to other areas studied. Heritability estimates of total brain, grey and white matter volume with exclusion of the two individuals with MMSE of 22 and their corresponding twin partners (0.902, 0.914 and 0.840, respectively) closely aligned with the values for the overall analysed group.

Differences in heritability based on laterality varied across the lobes. Heritability of the right brain hemisphere (0.881) closely resembled that of left brain hemisphere (0.861) with overlapping confidence intervals, and this similarity was also observed for volume measurements of the right (0.870) and left (0.869) cerebrum, and right (0.806) and left (0.831) parietal lobes. Several areas demonstrated stronger heritability on the left side compared to the right, including the frontal lobe (L 0.862, R 0.749) and temporal lobe (0.920, 0.870). The brainstem (R 0.755, L 0.693) and cerebellum (0.773, 0.721) had higher results for heritability on the right side compared to the left.

### Influence of gender and age

The differences in the additive genetics pathway (A) suggested that heritability may generally be slightly higher in males, and in many areas may decrease with age (see Table 4), however the majority of path estimates involved a 95% confidence interval crossing zero.

## Discussion

Previous twin studies have reliably demonstrated high levels of heritability for brain volume measurements on MRI in paediatric and adult twin populations [13–23]. Few studies have measured the heritability for brain volumes in middle to advanced age, and to our knowledge, no previous publications have studied an East Asian twin population in this age group. Our study aimed to map global and lobar brain volume heritability in middle to advanced age Japanese twins using classical twin design and structural equation modeling.

### Intracranial volumes

The volumetric analyses performed in this study produced mean brain volumes similar to those in other studies of East Asian participants of a similar age group [4, 5, 29]. The participants in the study by Chee et al. were of similar average age (65.8) to our cohort and had similar mean volume measurements to our study subjects. For example, total cerebral volume was 873.54cm^3^ compared to 892.7cm^3^ in our study. The volume measurements obtained for the group as a whole were also similar to those in studies of non-Asian participants when age is considered [6, 13, 21].

As the brain ages, brain volume decreases and CSF volume increases [7, 8], and our results were consistent with these well-established relationships. The correlation coefficient for total brain volume and age in our study was -0.42, which is similar to that found in other studies such as -0.33 by Batouli et al. [21] and the general trend of decreasing brain volume with age [4, 29]. In our study, white matter correlation coefficients were smaller in magnitude, for example total white matter (-0.047), than several previous studies of older twins [3, 21]. The difference may reflect ethnic variation, or be due to the wide and older age range in our twin group since brain atrophy is known to accelerate with advancing age [4].

### Heritability estimates

Our study found that genetic effects, as opposed to environmental, strongly determined total brain (90.2%), grey matter (91.2%), white matter (84.3%) and lobar volumes in middle to advanced age. The results for global brain volume heritability in our study are generally slightly higher than those in previous studies. A recent study by Batouli et al. [21] of twins of average age 71.4 years measured heritability of total brain, grey matter and white matter volume as 64%, 68% and 71% respectively. A study by Baare et al. in 2001 [13] estimated the heritability of total brain volume to be 90%, grey matter 82% and white matter 88%, with an average age of participants around 30 years and a methodology involving siblings as well as twin pairs. A recent study by Bohlken et al. [30] that mainly evaluated the heritability of structural brain network topology estimated the heritability of white matter to be as high as 96%. Other estimates of total brain volume heritability are 46% [14], 64% [15], 73% [16] and 66% [31] in studies involving different age groups and methodologies, indicating the wide range of estimations in published literature.

The results from our study indicate spatial variability in brain volume heritability across lobes and hemispheres. Our heritability findings for the frontal (86.7%), temporal (92.3%), parietal (86.5%) and occipital (64.3%) lobes are slightly higher but comparable to those calculated by Panizzon et al. 2009 [32] in their study of mean age 55 year old twins, where heritability for frontal, temporal, parietal and occipital lobes were approximately 85%, 86%, 82% and 48% respectively. By contrast, heritability estimates by Geschwind et al. in their study of mean age 71 year old twins were 54%, 46%, 47% and 28% for the same four lobes respectively. These two studies and ours commonly found that the occipital lobe had the weakest genetic contribution of the four lobes. In the recent study by Batouli et al. [21] heritability estimates of 64%, 55%, 63% and 67% were produced for these same four lobar volumes respectively. Cerebellar volume heritability was estimated as 67% in the study by Batouli et al, 69% in a study of paediatric twins by Yoon et al [18] and 81% in a study of average age 32 years adults by Posthuma et al. [33], compared to our study estimate of 84.6%. Brainstem volume heritability, to our knowledge, has not been previously studied in this age group so our data may represent the first estimation of genetic influence.

Differences in heritability estimates may be due to a number of factors. To our knowledge, this is the first study to examine global and lobar brain volume heritability in middle to advanced age East Asian twins. Therefore the differences observed in heritability compared to previous studies may reflect ethnic variation. Our study involved a twin group with a mean age of 62.5 (range 41 to 85) years where as some studies involved adult twins with higher mean ages, such as 71 [15] and 71.4 years [21], and others lower, such as 55 [32] and 30 years [13]. This may explain some of the differences since heritability of brain volume has been observed to vary with age [3, 21, 24]. Differences in methodologies may also explain some of the variation in heritability estimates since some studies [11, 21] used AE models, where as other publications [15, 18, 23, 33] used ACE models, where a pathway for common environmental factors (C) is also included, potentially decreasing the heritability (A) pathway estimates. Furthermore, previous studies have included siblings [11, 14, 33] or non gender matched twin pairs [11, 13] in their study group, where as our study involved only monozygotic and dizygotic gender matched twins in the final estimations of heritability. Lastly, the images used in our study were obtained on a 3T MRI and were 3D T1 weighted, high-resolution 1.0x1.0x1.0mm and high contrast. Previous studies have used lower resolution images such as Panizzon et al. [32] with 1.3x1.0x1.3, or Batouli et al. with 1.0x1.0x1.5 [21], and many previous studies have used a 1.5T [15, 18, 21] MRI. This means that the images in our study may have provided more accurate volume measurements, particularly for lobar volumes or areas where atrophy is present, which may explain the differences observed in heritability estimates.

### Heritability and age, gender and laterality

Global and lobar brain volume measurements examined in our study demonstrated decreased heritability with the age covariate and this relationship has been observed in previous studies [21, 24]. Our finding that the heritability of global and lobar brain volume measurements is possibly higher males compared to females is also supported by a number of previous studies [21, 33]. Heritability estimates that appear to be most influenced by gender included the left hemisphere, left cerebrum, left cerebellum and frontal lobe. The study by Batouli et al. also observed a relatively strong influence of gender for the heritability of the frontal lobe and left cerebellar volume, but not for the left hemisphere or cerebrum where the influence was moderate.

In our study, the estimated heritability of the right hemisphere (88.1%) was slightly higher than the left (86.1%), with overlapping confidence intervals. However, several previous studies [18, 21, 23] found that the left side had the stronger genetic influence, and others [15] have suggested hemisphere heritability may be influenced by handedness, which was not assessed in our participants. Furthermore, in our study the gender and age covariates appeared to have a stronger influence on left hemisphere volume compared to right, which may explain the lower result for left hemisphere volume heritability. Heritability on the left side for the frontal, temporal and parietal lobes was slightly higher when compared to the right; a finding consistent with the study by Yoon et al. of paediatric twins.

### Study limitations

Our study had several limitations. Firstly, our sample size for the final results was 72 twin individuals, which is similar to some previous studies [18, 24], but smaller than other studies with over 200 subjects [16, 21, 23]. Furthermore, whilst participants were selected to satisfy an age requirement, the age range (41 to 85 years, mean 62.5) was wide, and brain heritability may greatly vary within this age range. Many diseases associated with brain atrophy on MRI, such as dementia, have a peak age of incidence greater than 62.5 years. Due to equipment changes at the department, the twin group were scanned on two different 3T MRI machines, meaning there may have been variation in the images and then subsequent volume measurements based on the scanner. Acknowledging this, all twin pairs were scanned with the same machine on the same day, and the machine was included as a covariate in the structural equation models used to produce the final heritability estimates.

Two individuals in our analysed sample recorded MMSE scores below 24, introducing possible bias from inclusion of individuals with cognitive impairment. The typically used MMSE criteria for ‘normal’ cognitive function is usually a score of 24 or greater, although some authors have suggested that lower scores may be normal depending on other factors (e.g. education level) [34]. To estimate the effect of inclusion of these individuals with MMSE score below 24 in our analysis, we repeated the intra-pair correlations and structural equation models for heritability with these two individuals and their corresponding twin pairs removed, and the values closely aligned to those for the overall analysed group.

There are inherent limitations that come from twin studies using structural equation modelling. The approach used in this study to produce the final heritability estimates for the majority of volume measurements was an AE model (A = additive genetics, E = unshared environmental effects), selected based on AIC and log-likelihood ratios. The AE model includes two phenotype variance components and is a commonly used model for twin studies, however it does omit the “common environmental effects” pathway, which may slightly exaggerate heritability estimates.

## Conclusion

This study calculated the heritability of global and lobar brain volumes on MRI in middle to advanced age Japanese twins using classical twin analyses and structural equation modelling.

Total brain, grey matter and white matter volumes were confirmed to decrease with age, and found to have strong heritability (90%, 91% and 84% respectively), and lobar volumes also demonstrated high heritability in middle to advanced age. Genetic influence may be greater in males and decrease marginally with age. The heritability of global and lobar brain volumes is possibly higher than previous estimates and higher in adults of East Asian ethnicity.
